# Building confidence in crises – the roles of Sierra Leonean religious leaders’ during the 2014–2016 Ebola outbreak

**DOI:** 10.1080/16549716.2025.2555046

**Published:** 2025-09-19

**Authors:** Padraig Lyons, Maike Winters, Mohamed F. Jalloh, Helena Nordenstedt, Helle Mölsted Alvesson

**Affiliations:** a Department of Global Public Health, Karolinska Institutet, Stockholm, Sweden; b Department of Health and Human Service (HHS), Centers for Disease Control and Prevention, Atlanta, GA, USA

**Keywords:** Ebola virus disease, burial practices, religious leaders, risk communication, Sierra Leone

## Abstract

**Background:**

Religious leaders have been involved in health promotion campaigns for many years across West Africa, such as their engagement in the HIV/AIDS pandemic response. This involvement in public health campaigns has indicated varied results in the past and therefore a critical approach is required when engaging them in the outbreak response. Little is known about religious leaders’ perceptions of their role in communicating Ebola risks during the 2014–2016 outbreak in Sierra Leone.

**Objectives:**

To better understand religious leader’s perceptions of their roles communicating risk during the Ebola outbreak in Sierra Leone.

**Methods:**

In this qualitative study, 10 semi-structured interviews were conducted with religious leaders in Freetown, Sierra Leone. Five Christian and Islamic leaders were recruited from multiple national religious organizations including male and female leaders. Data were analysed using thematic analysis.

**Results:**

Three themes were developed from the data that illustrated the different strategies religious leaders used when establishing public confidence in their role communicating risk and the messages they communicated during the outbreak. Religious leaders described how they established themselves as non-political actors in the outbreak response. Religious leaders both adapted pre-existing roles, including offering community support during crises, and assumed new responsibilities, such as fostering interreligious collaboration to develop Ebola-specific risk communication strategies.

**Conclusions:**

Religious leaders were pragmatic in their approach to risk communication, leveraging political distrust and collaborating with other actors to strengthen their position. Interreligious unity and scripturally supported messaging helped to establish confidence in the public health emergency response.

## Paper context

- **Main findings:** Religious leaders were pragmatic in their approach to risk communication, leveraging political distrust and collaborating with other actors to strengthen their position using interreligious unity and scripturally supported messaging to establish confidence in the public health emergency response.
- **Added knowledge:** In their role communicating risk religious leaders prioritized building confidence by advocating for community supported Ebola-measures and by establishing themselves as non-political actors in the outbreak response.
- **Global health impact for policy and action:** Public health messages developed by religious leaders can be used as part of risk communication campaigns to improve their public acceptability.

## Background

Across the world, religious leaders are trusted figures in their communities, and as such, can be positive agents of health behaviour change through their involvement in public health interventions [1–4]. For instance, hearing messages from religious leaders such as pastors or other ministerial staff that were supportive of same-sex relationships was associated with an increased willingness to use HIV prevention strategies (e.g. pre-exposure prophylaxis) among Black Americans in the United States [5]. In Ghana, an intervention leveraging national and local religious leaders to disseminate HIV public health messages via mass media was associated with reduced HIV-related stigma [6].

Religious leaders have also been associated with hindering public health efforts [7]. In the early 2000s in Northern Nigeria, religious leaders propagated misinformation that the oral polio vaccine contained the HIV-virus and antifertility agents. This misinformation contributed to the suspension of the polio vaccine campaigns in the region [8]. During the HIV/AIDS epidemic, some religious groups in Nigeria and Burkina Faso opted to promote abstinence over condom use [9,10], whereas others actively encouraged condom use to reduce the spread of infection [11]. The mixed messages also reflected the influence of religious leaders; they have been both positively and negatively linked to health and social outcomes in the HIV/AIDS epidemic [6,9,10,12,13]. Religious leaders also have ability to influence vaccine uptake as was demonstrated during the COVID-19 pandemic. In Romania for example, the strong standing of religion combined with negative attitudes towards vaccines held by some influential religious leaders is thought to have contributed to low vaccination coverage [3]. In Israel, on the other hand, where vaccination coverage was very high, religious leaders were acknowledged for playing a key function in community engagement around COVID-19 vaccination [14]. Nevertheless, drawing a causal link between religious influence and health outcomes is difficult, despite well-established associations.

Religion and religious identity are important facets of Sierra Leonean society. The majority of Sierra Leoneans are affiliated with either Islam (77%) or Christianity (22%) [15]. Countrywide, religious leaders are involved in various public health campaigns through faith-based organizations such as the Islamic Action Group (ISLAG) and the Christian Action Group (CHRISTAG). During the civil war in Sierra Leone (1991–2002) ISLAG and CHRISTAG were brought together to form the Inter-Religious Council of Sierra Leone (IRCSL), an umbrella entity responsible for religious leaders’ coordination and advocacy related to wide-ranging societal matters [1]. The IRCSL facilitated dialogue between the Government of Sierra Leone and rebel groups that contributed to a peace agreement that eventually ended the civil war in 2002 [16,17].

The Ebola epidemic posed a unique religious challenge in Sierra Leone: religious and cultural practices (such as washing a dead body before burial) were strongly associated with Ebola transmission [18,19]. In several West African countries, including Sierra Leone, washing (or ablution) and dressing of a deceased family member in preparation for burial are important religious and cultural traditions [20]. Traditional burial practices were major sources of Ebola transmission linked to a number of case-clusters and researchers have estimated that 2.5 new cases of Ebola resulted from every unsafe burial performed [19,21–23].

In 2014, the World Health Organization (WHO) published updated protocols for the safe and dignified burial of corpses during the outbreak [24]. Specialized burial teams were established by WHO to handle burials in a safe, supervised and controlled manner. Safe and dignified burial protocols eventually incorporated feedback from religious leaders who provided guidelines for culturally appropriate modifications to traditional burials [24,25]. In collaboration with public health professionals, religious leaders developed scripturally based messages that were delivered nationwide in places of worship and local community events as well as through radio and television programmes [26].

To date, there is a growing body of research examining the role of religious leaders as risk communicators during health emergencies, including in Sierra Leone’s Ebola outbreak. For instance, previous research has shown that public health messages promoted by religious leaders were positively associated with willingness to engage in safe burial practices during the Ebola outbreak in Sierra Leone [27]. In-depth qualitative understanding of the religious leaders’ own perspectives on their role in health emergencies remains limited. This study aimed to examine and contextualize the strategies adopted by religious leaders to communicate risk during the Ebola outbreak in Sierra Leone.

## Methods

### Setting

This study was carried out in Freetown, the capital of Sierra Leone where approximately one million of the country’s eight million people live. Sierra Leone is a low-income country situated in West Africa. Its economy was recovering from a brutal decade-long war when the country was struck by the devasting Ebola outbreak from 2014 to 2016. The country has one of the highest maternal mortality rates and had a population with a life expectancy of 60 years in 2021 [28]. Freetown was one of the worst affected areas during the outbreak with a high number of positive cases and deaths [29]. Participants in this study lived and worked in Freetown at the time of interview. This study was conducted in collaboration with FOCUS1000, a local nongovernmental organization (NGO) that facilitated the engagement of religious and other community leaders during Sierra Leone’s Ebola outbreak.

### Study design & sampling methods

This qualitative study was based on semi-structured interviews with religious leaders that were involved in risk communication campaigns across Sierra Leone during the Ebola outbreak 2014–2016. Ten religious leaders who met the criteria for inclusion in the study were recruited by a trained peer. Religious leaders were selected using purposive sampling, with predefined criteria that all participants must have been involved in risk communication activities during the outbreak. Quota sampling, a form of purposive sampling, was used to attain equal numbers of Christian and Islamic religious leaders while also ensuring a balanced representation of female religious leaders. FOCUS1000 assisted in recruiting the participants through existing networks, including IRCSL, ISLAG and CHRISTAG. The sample of 10 participants comprised equal numbers of Christian and Islamic religious leaders. Participants were predominantly male (*n* = 7). Five participants were pastors, three were imams, and two were Islamic leaders with executive leadership roles in umbrella organizations during the Ebola outbreak. Functional characteristics of the participants in the study are described in Table 1.

**Table 1.** Participant information.

Interview ID	Gender	Interview length (Mins)	Religious background	Interview location
Interview 1	Male	57	Islam	FOCUS1000
Interview 2	Male	69	Islam	FOCUS1000
Interview 3	Male	43	Islam	Mosque
Interview 4	Male	83	Christian	FOCUS1000
Interview 5	Female	25	Islam	FOCUS1000
Interview 6	Male	70	Christian	FOCUS1000
Interview 7	Male	48	Christian	FOCUS1000
Interview 8	Female	74	Christian	Pastor’s home
Interview 9	Male	57	Christian	FOCUS1000
Interview 10	Female	33	Islam	FOCUS1000

### Data collection and analysis

The interview guide used in the study was informed by the WHO guidelines for communicating risk in public health emergencies, which also provided a framework for analysing the data [30]. Questions were designed chronologically, asking questions related to experiences before, during, and after the outbreak. The interview guide comprised open-ended questions regarding the participants’ religious background and previous risk communication experience, Ebola knowledge, and experiences of barriers and facilitators in conducting risk communication campaigns during the outbreak. Finally, open-ended questions were posed regarding reflections on past experiences and recommendations for the future. Two pilot interviews were conducted with two of the religious leaders included in the study, leading to minor adjustments to question wording and ordering, before finalizing the guide for the remaining interviews.

All interviews were in-person and conducted in English. Although interviewees all spoke Krio, the lingua franca in Sierra Leone, they were also fluent and comfortable conducting the interviews in English as confirmed during the recruitment. Moreover, given that the interview guide was developed in English, conducting the interviews in English was important for preserving meaning and avoiding translation and back-translation whereby meaning could be lost. All 10 participants were interviewed between 5 March and 20 March 2019 in Freetown, Sierra Leone. Eight interviews were conducted at the headquarters of FOCUS1000, one took place in a local religious school, and one was conducted in a participant’s home as per their preference. Interviews were conducted by a single interviewer, who was not associated with the organizations in Freetown, Sierra Leone. No one else was present during the interviews. Each interview lasted 56 min on average, ranging between 25 and 83 min (Table 1).

Interviews were transcribed verbatim and thematically analysed using Dedoose web-based software, version 8.3.4. The thematic analysis began with an initial coding of three of the transcripts using both in vivo codes and codes derived by the lead author from the text. This coding framework was then applied across the remaining seven interviews. When all transcripts were coded, several emerging codes were retrospectively applied across the interviews and those first transcripts were revised again to check for consistency in the coding process. The codes were grouped into categories leading to the formation of sub-themes and final themes. During the analysis, authors discussed and provided feedback on codes and themes were developed. In examining religious leaders’ responses to one another, the analysis became more latent allowing for a deeper understanding of their opinions on their work during the outbreak.

### Ethical considerations

The Sierra Leone Ethics and Scientific Review Committee granted ethical approval for the study on 28 February 2019. There is no linked ethical approval number although the signed and approved form can be provided upon request. Prior to participation in the study, all interviewees provided informed consent to participate by signing the informed consent form. This study has been performed in accordance with the principles stated in the Declaration of Helsinki.

## Results

Three primary themes were identified from the data, reflecting the strategies religious leaders used to enhance public confidence in their role as risk communicators and the messages they communicated: (1) establishing themselves as trusted non-political actors (2) providing community support in the context of an Ebola outbreak, and (3) collaborating via interreligious platforms and with other actors in the Ebola response. The religious leaders discussed these three themes as strategies they flexibly used to build confidence in their role communicating risk during the outbreak. The religious leaders adapted pre-existing roles such as providing community support in times of crisis and established new ones to build public confidence for the recommended public health practices during the outbreak. Themes are categorized and summarized in Table 2. They are further discussed and illustrated with quotes below.

**Table 2.** Categories, sub-themes and themes.

Religious leaders are trusted because they are ingrained in communities and work in communities, unlike politicians	Religious leaders are set apart from politicians because they come from within communities and serve the people	**Establishing religious leaders as non-political actors**
People don’t listen to political people like they listen to religious people because they understand that when religious people speak it is going to be a message that benefits them.		
Messages from religious leaders are taken more seriously because people have more confidence in religious leaders than politicians		
Religious leaders have more public confidence than politicians because they can deal with ideologies	Religious leaders are not incentivized by money or other benefits the way politicians are	
People don’t trust politicians or medical people so religious leaders are seen as neutral people		
People don’t have confidence in politicians because they are paid for what they do, unlike religious leaders		
Advocating to the government to add a dignified aspect to Ebola burials	Advocating to authorities regarding burial practices	**Providing community support in the context of an Ebola outbreak**
Raising public requests to allow family members to attend Ebola victims’ funerals to authorities		
Raising public requests to have more women on burial teams to authorities		
Providing emotional and spiritual support to people in quarantine	Religious leaders provided support for those in quarantine	
Giving medical advice to people in quarantine		
Addressing public fears and concerns regarding burial teams	Religious leaders providing community support for other Ebola related issues	
Support figure for people in communities whose family died from Ebola		
Providing support to those who are sick in hospitals and at home		
Interreligious collaboration provides a believability and establishes public confidence in religious leaders messages	Interreligious collaboration helped to build confidence in the religious leaders as messengers during the outbreak	**Collaboration built public confidence in religious leaders**
Religious leaders speaking with one voice – prevents conflicting messages and increases trustworthiness of the messages		
Some religious leaders’ messages caused confusion early in the outbreak		
Collaboration with technical people regarding specific issues (medical people accompanying to answer medical questions) helps build public confidence in the religious leaders as risk communicators	Collaboration with technical people and local leaders built public confidence in religious leaders messages	
Collaboration with local leaders and traditional healers to build support in Ebola messages		

### Establishing religious leaders as trusted non-political actors

Early in the Ebola outbreak, political conspiracies and distrust in political figures were commonplace in Sierra Leone. Religious leaders described how they established public confidence as Ebola risk communicators by separating themselves from politicians and political organizations.

*…* [the public] don’t know whether to believe the health workers or whether to believe the politicians, so they were confused. So this made us [religious leaders] to be relevant in the message dissemination because they are seeing us as neutral people. (Interview 1 – Muslim Leader)

… you will discover that the religious leader will get more results than that politician because they [politicians] are coming from the angle of distrust. (Interview 4 – Christian Leader)

The religious leaders describe how their separation from political affiliations provided them with a unique platform to approach their risk communication efforts, distinct from politicians.

Some of them [the public] were saying it is good because they lack trust in the politician, but since you people [religious leaders] are here, we believe you. We will go by what you say. (Interview 4 – Christian Leader)

People within communities respected religious leaders when they communicated Ebola messages because, unlike politicians, they were frequently seen and embedded within communities.

… so that’s the big tool that we have that the politicians don’t have, even those in power. Because maybe he [Politician] is in this house [house belonging to a member of the public]. The people that are there, he doesn’t know them. A religious person doesn’t do that. I go there first thing in the morning saying ‘Good morning how is the old man, the old lady, are they okay?’ Maybe there is a problem that I need to sort out, to come to their aid. (Interview 2 – Muslim Leader)

One religious leader speculated that the reason political figures are not trusted like the religious leaders is because of the financial gain involved in their political roles.

When the minister [political leader] talks, he is lying. But when I appear on TV … they [the public] know when I say something it is going to work, it is going to benefit them. Because they [the public] don’t pay me for this work … I don’t have a new bicycle, but I’m dedicated to doing this job. (Interview 2 – Muslim Leader)

The religious leaders define their work as vocational and their lack of apparent wealth as a reason to garner public confidence and trust.

### Providing community support in the context of an Ebola outbreak

As a result of the direct and indirect effects of Ebola, many Sierra Leoneans found themselves in challenging situations. The religious leaders described themselves as figures of hope for their communities in many of these circumstances, providing a listening ear and spiritual meaning to the situations the public faced.

… we give them the words of hope, in the messages, give them courage that they will not die, they will come out. Let them just listen to what the doctor say. Follow the instructions … . Sometimes they call me 2, 3 am in the morning; ‘reverend I am giving up, the pain I am feeling so much pain, even to hold the cup for me to drink I cannot, the pain’. I said ‘This is not for you. You will surely come over this’. (Interview 8 – Christian Leader)

This *hope* the religious leaders described was contextualized in terms of the individual’s spirituality and the essence of placing faith in a higher power beyond the outbreak. The religious leaders described how they stood outside the homes of those who were quarantined and reassured them that they will recover from the emotional and potentially physical burden placed upon them by the outbreak.

The suffering community members were facing during the outbreak was contextualized by the religious leaders who compared Ebola to the infectious diseases discussed in the Bible (leprosy) and the Qur’an (the *Tha’oon Amwas*). This facilitated public understanding of the Ebola measures that religious leaders were communicating while also providing a means to spiritually interpret the outbreak.

There’s nothing in Islam called Ebola. But there are similar things, like the tha’oon [infectious disease discussed in the Qur’an] (Interview 2 – Muslim Leader)

In the bible lepers were set apart … because they thought it was a contagious disease (Interview 9 – Christian Leader)

The religious leaders further reported building confidence in their messages by advocating for the needs of their communities. Religious leaders describe how they worked with health authorities to design safe and dignified burials, including incorporating more women into burial teams in response to concerns that deceased women were being buried by men.

The burial team was full of males. So, at that time, the women, they rose up and they said uh no! We need women in the burial teams. We went to the government and we said this is the concern of our people … .The government agreed. (Interview 4 – Christian Leader)

The religious leaders described this advocacy as a tool to provide their communities with what they needed at a time of crisis and in doing so built trust in their role as communicators.

[the public] want to appeal to you, to appeal to the government in the area of burial … even though they are talking about safe burial, we want to add dignity into it. It is our culture, we need to give our funeral rights to our loved ones when they die. (Interview 8 – Christian Leader)

### Collaboration built public confidence in religious leaders

During the Ebola outbreak, Christian and Islamic leaders worked together to develop and communicate Ebola messages across Sierra Leone in a coordinated manner. The religious leaders described how this interreligious collaboration was beneficial for the risk communication campaigns as it emphasized, for the public, the importance of putting aside religious differences to focus on the outbreak.

At first, they [the public] were thinking that because of our different beliefs it would be difficult for us [Muslims and Christians] to come together, you know, but we were able to make the people know that, no, in fact, it is the source of our strength to come together … Some of us were able to say look, when Ebola is coming it is not looking for a Christian, it’s not looking for a Muslim, it’s not looking for your tribe, it’s not looking for your colour, it is just looking for an individual that it can suppress, kill, destroy. (Interview 4 – Christian Leader)

The religious leaders described the importance for the public to see Islamic and Christian leaders entering towns and villages together. They described how standing side-by-side helped the public to better appreciate the importance of their messages.

We do everything in common. Sometimes we even go into churches, when it is required to go to the church, we go to the church when it is required to come to the mosque, we do that also. Sometimes you can see some Christians in the mosque, sometimes you can see some Muslim in the church. Just for the people to get the message. (Interview 1 – Christian Leader)

This interreligious collaboration was described from a more pragmatic perspective by several religious leaders. They acknowledged the confusion caused by different, often conflicting messages from multiple sources during the outbreak. They considered religious leaders of different doctrines speaking with one unified voice to be much clearer and more inherently understandable to their audiences.

… you know initially, at first, the misinformation was so much … the information was coming in every direction … .everyone was confused. (Interview 6 – Christian Leader)

… we said that we are going to speak with one voice. We don’t want to speak with conflicting messages. So that at least when you go to the south, I go to the north and he goes to the east, we make sure that the message that you are giving the people is the same message. Don’t say any different thing. So, we all speak with one voice. Yes, no conflicting message. (Interview 7 – Christian Leader)

There was acknowledgement from religious leaders that they contributed to the public’s confusion earlier in the outbreak. The subsequent unified approach to risk communication was described as important to prevent the spread of false information by religious leaders.

… messages came that people should wash [their body] with water and salt [solution], then the Ebola will not affect you, oh yeah … a prophet from Nigeria, you know, they gave a potion… it went [around] like a wildfire. Even Muslims were washing, they were bathing with that stuff. (Interview 4 – Christian Leader)

Collaboration was also described by religious leaders in terms of their interactions with other sectors of the Ebola response and the beneficial impact this had on their work overall. When communicating their messages, religious leaders often entered communities as part of a larger team which included medical experts and other non-religious people. The presence of religious leaders beside these medical teams made their messaging more acceptable to the public.

When it comes to the technical parts of the disease, we call on the technical people. Because when we are going [into communities] sometimes we go with some health officers, we involve the DHMT [District Health Medical Team]… We will call them to come on board and they explain to the people. But because we are around, they [public] will accept them [medical team messages]. (Interview 4 – Christian Leader)

The religious leaders would also engage village chiefs, community leaders and traditional healers upon entering towns and villages to support their communication campaigns. These local leaders would help to disseminate messages through communities using megaphones and going to house to spread the messages. The religious leaders also described their approach to collaboration in pragmatic terms.

We also saw it necessary to work with the traditional healers because the practice of the traditional healers very [much] contradicts the belief of the Christian and the Muslim. But because it was a national emergency the thing [Ebola] was killing our people, we had to say no, let’s ignore some of this … fundamental right … and then address the issue that is killing people. (Interview 4 – Christian Leader)

Although they may not have agreed with external parties such as the traditional healers, the religious leaders expressed willingness to put this aside for the greater good as a practical modification to their standard practice prior to the Ebola outbreak.

## Discussion

Three primary strategies were leveraged by religious leaders to build confidence in their role communicating risk and the messages they communicated. First, they established themselves as trusted non-political actors in the outbreak. Second, they provided community support that went beyond just disseminating health messages. Finally, they collaborated via their inter-religious platforms and engaged with other actors in the outbreak response to build confidence in their communicated messages.

Religious leaders in this study describe how their perceived separation from political affiliations provided them with a unique platform to approach their risk communication efforts during the outbreak. Religious leaders are trusted figures in Sierra Leonean society, particularly by those with relevant religious affiliations, partly due to their involvement with the IRCSL in facilitating peace talks during the civil war in Sierra Leone [31]. More broadly, the central role of religion in Sierra Leonean society including involvement in health promotion and humanitarian work also laid a foundation for religious leaders’ engagement during the outbreak [31]. On the other hand, according to religious leaders, politicians and others holding leadership positions in Sierra Leone were not seen as widely trusted due to factors such as their perceived role in the civil war and the perception of government elites financially benefiting from the emergency [32,33]. Similar feelings of mistrust have been described during Ebola outbreaks in Democratic Republic of Congo (DRC) in recent years. Mistrust of local elites has been linked to the influx of donor aid money and the latent suspicions that powerful actors are profiteering from ongoing crisis [34].

During the outbreak in Sierra Leone, there is evidence that traditional authorities, such as village chiefs, were not always trusted by members of their communities as purveyors of Ebola-related information due to complex post-colonial relations with the state and international actors [32]. The findings in our study suggest that religious leaders built confidence in their messages by distancing themselves from politicians, who they viewed as not widely trusted by the public [35]. The strategy of religious leaders distinguishing themselves from politicians may have been effective in building public confidence and modifying risky burial practices, but it was not possible to discern such effects with the available data in this study. In a separate national study, people who received public health messages promoted by religious leaders during Sierra Leone’s Ebola outbreak were more willing to engage in safe burial practices such as avoiding direct physical contact with corpses [27].

The importance of building public confidence and trust in the messenger is key to effective risk communication as has been established previously using the socio-ecological model [36–38]. The leaders described how they did not receive financial benefit from their involvement in the outbreak unlike their political counterparts, which they believed enhanced their credibility and trust by the public. Instead of associating with political leaders, the religious leaders sought different trust alliances such as through inter-religious collaborations. Outside of Sierra Leone, religious leaders in Israel have highlighted how political distrust during the COVID-19 pandemic posed a challenge to their position in the outbreak response, feeling ‘stuck’ between the policies of government institutions and the needs of the public [39]. Research examining levels of public trust in Ebola treatment centers at the time of the Ebola outbreak also found that low levels of trust in the treatment centers early in the outbreak compromised the public’s willingness to seek care from such facilities. Public confidence in treatment centers improved as the outbreak progressed thanks in part to positive endorsements by survivors and families who had members taken to the facility – highlighting the importance of establishing public trust via trusted messengers during the outbreak [40].

Numerous religious leaders referred to themselves as *non-political* and *neutral* when describing their role in Sierra Leonean society. While they pragmatically separated themselves from political affiliations to assist in their risk communication strategies, evidence suggests that religion and politics are closely intertwined and difficult to separate from one another in Sierra Leone [41,42]. The religious leaders in the study did not organically share their reflections on the entanglement of religion and politics when referring to their role as non-political and neutral. Religious leaders themselves acknowledged that in the beginning of the outbreak they wrongly promoted risky health behaviors but this was before they were educated on the correct practices.

While Sierra Leone is unique with its high levels of interreligious tolerance, the power of communicating with a unified message holds relevant lessons for other emergency settings. Although the leaders in this study did not discuss the ideological conflicts that can exist between the two religions [43], the results highlight the importance of finding a middle ground to help bolster a collaborating position focused on a unified goal. This is also shown through the religious leaders’ collaboration with traditional healers, for which doctrinal differences were put aside to fight their common proverbial enemy [36,44]. Religious leaders involved in the COVID-19 response in Israel describe a willingness to set aside traditional religious practices during the pandemic and reflect on the challenges this posed, for example, advising against congregating to pray or attend family or religious events [39]. In DRC, faith leaders were similarly involved in public health campaigns during COVID-19, but they expressed dissatisfaction with the lack of formal training to inform their health response work and voiced a desire to be involved in the co-development of response messages to better facilitate their public communication [45]. Beyond Ebola and COVID-19 responses, religious leaders have shown a willingness to engage in promoting public health messaging although to varying degrees depending on the topic in question [46]. It has been suggested that the involvement of high-level religious leaders, with higher levels of education and experience merging their religious with scientific views, can be influential opinion leaders to lower-ranking, community-based religious leaders [46].

Religious leaders were embedded in communities and could appreciate the specific needs of individual community members while also distributing messaging through wider communication campaigns. The socio-ecological framework highlights the importance of intrapersonal and interpersonal dynamics when trying to affect behaviour change [47,48]. The top-down approach to communication seen early in the outbreak was not as effective in part because it did not account for the socio-cultural realities of many Sierra Leoneans, including lack of adequate considerations for incorporating dignified modifications to safe burial practices. Moreover, there were limited considerations for educating the population on how to safely care for sick family members while waiting for medical help to arrive, which was usually severely delayed and challenging in the earlier phase of the response [1,49]. It has been noted elsewhere that asking community leaders to reiterate standard messaging can discredit their legitimacy in the eyes of the public if doing so does not align with local expectations or priorities [50]. It is important to consider that religious leaders may not always be inherently trusted by their communities particularly in contexts where they may be perceived as not representing the best interest of their community members, especially if what they promote may be viewed as incompatible with societal or religious values. Nevertheless, engaging religious leaders early in the process is likely to have important benefits for how they navigate these practical realities of their own trustworthiness and religious authenticity, balanced against the public health goals of their adopted risk communication role during future health emergencies.

Religious leaders willingness to engage with health professionals and develop Ebola specific messages from religious texts may be a practical and beneficial application of the findings of this study to inform similar future approaches during health emergencies. The importance of building public confidence and trust in the messenger is equally important as the messages developed to communicate risk [36–38]. Additional research is needed to understand the comparative trust and effectiveness of various categories of community-level messengers in how they communicate risk and promote protective health measures during health emergencies.

### Strengths and limitations

A strength of this study is that it provides in-depth insights from religious leaders, Christian and Islamic, who had an active role in risk communication during the 2014–2016 Ebola response in Sierra Leone. To the author’s knowledge, no prior study has assessed the perspectives of these religious leaders in relation to their risk communication role during the outbreak. However, the study is also subject to several limitations. For example, the study sample is limited to religious leaders from the capital city, Freetown, most of whom held powerful positions in umbrella faith-based organizations in Sierra Leone. As such, their opinions may not be necessarily transferable to all religious leaders in Freetown or other parts of Sierra Leone, especially more rural areas. For instance, all 10 participants spoke English fluently, which may not be true for many other religious leaders in the country. Nonetheless, the perspective of these leaders may provide a more overarching viewpoint of religious leader involvement in the outbreak, though it was difficult to discern their social desirability bias to reinforce their religious influence and relevance during health emergencies. While the sample was limited to 10 religious leaders, the power could be considered robust since the aim of the study was specific to religious leaders working in leadership roles in faith-based organizations during the outbreak [51].

The study was subject to other potential sources of bias. Interviews were carried out by PL who is a caucasian male foreigner, which may have introduced a response bias, though difficult to discern. It is plausible that the religious leaders may have provided more candid and contextually nuanced responses to a Sierra Leonean interviewer. On the other hand, they may have provided the most honest answers to someone they perceive as an outsider, particularly when criticizing the outbreak response efforts – something they may have hesitated to do if the interviews were conducted by a Sierra Leonean. Harvey’s ‘elite interviews’ also helped to inform PL on how to approach the interviews in terms of building trust and engaging these religious leaders who are considered high standing community figures [52]. The interviews were designed with a combination of open-ended and closed questions with freedom in terms of duration and questions type to allow interviewees to provide responses in a combination of ways as Harvey described. An unavoidable source of bias in this study was that the qualitative interviews were conducted more than 3 years after the outbreak, which might have led to omission of granular details and possible recency and recall bias. The risk of recall bias was mitigated in part by encouraging the leaders to describe their participation chronologically to trigger their memory of details. Participants were all living in Freetown during and after the Ebola response. Eight out of 10 interviews were conducted in FOCUS1000 headquarters, potentially leading to a response bias. Religious leaders’ involvement in the Ebola outbreak was coordinated by FOCUS1000; therefore, participants may have felt unable to criticize aspects of their involvement in the pandemic without compromising their position for future health promotion and community engagement work. This effect was mitigated by reiterating that the lead investigator was not affiliated with FOCUS1000 and all interviewees’ responses would remain entirely confidential.

## Conclusion

Religious leaders viewed themselves as key actors in risk communication efforts during the Ebola outbreak in Sierra Leone. Religious leaders in this study highlighted the key role that inter-religious collaboration and collaboration with public health officials had in their role communicating risk. They were pragmatic in their approach to risk communication and engaged with multiple different facets of the outbreak response to strengthen their position as trusted messengers. Preexisting umbrella networks allowed religious leaders to engage in scaling up risk communication strategies nationally during the Ebola response in Sierra Leone. They adapted practical strategies, such as intentionally differentiating themselves from politicians, to be seen as trusted and neutral parties who are not entangled with financial conflicts of interests. Religious leaders acknowledged their initial negative contributions to unsafe risky practices earlier in the outbreak before they were engaged, but equally demonstrated practical adaptability as reflected in their willingness to eventually adopt and promote protective practices after they were engaged. This finding points to the importance of early engagement of influential leaders, including religious leaders, during health emergencies – even if it is difficult to predict if their influence will produce the intended protective public health effects.

## Supplementary Material

Supplemental Material

COREQ_checklist_PL.docx

## Data Availability

The datasets used and/or analysed during the current study are available from the corresponding author upon reasonable request.
