# Supplementary material for: Building confidence in crises – the roles of Sierra Leonean religious leaders’ during the 2014–2016 Ebola outbreak
Source: Glob Health Action. 2025 Sep 19;18(1):2555046. doi: 10.1080/16549716.2025.2555046 (PMC12451959; doi:10.1080/16549716.2025.2555046)
Supplement: Supplemental Material [file ZGHA_A_2555046_SM9306.pdf]

GOVERNMENT OF SIERRA LEONE
Office of the Sierra Leone Ethics and Scientific Review Committee
Directorate of Training and Research
5th Floor, Youyi Building Brookfields, Freetown
Ministry of Health and Sanitation

28th February, 2019

To: Dr Padraig Lyons (Masters Student) **Principal Investigator**
Department of Public Health Sciences
Karolinska Institutet, Stockholm
Sweden
padraig.lyons25@gmail.com

Study Title: Religious Leaders as Communicators of Risk Information in the Ebola Outbreak in Freetown, Sierra Leone

Version: January, 2019

Supervisor **Assistant Professor Helena Nordenstedt**
Global and Sexual Health Research Group
Karolinska Institutet, Stockholm
Sweden
helena.nordenstedt@ki.se

Local Collaborator: Focus 1000

Submission Type: First protocol version submitted for review

Committee Action: Expedited Review

Approval Date: 28 February, 2019

The Sierra Leone Ethics and Scientific Review Committee (SLESRC) having conducted an expedited review of the above study protocol and determined that it presents minimal risk to subjects, **hereby grants ethical and scientific approval for it to be conducted in Sierra Leone.** The approval is valid for the period, **28 February, 2019– 31 December, 2019.** It is your responsibility to obtain re-approval/extension for any on-going research prior to its expiration date. The request for re-approval/extension must be supported by a progress report.

GOVERNMENT OF SIERRA LEONE
Office of the Sierra Leone Ethics and Scientific Review Committee
Directorate of Training and Research
5th Floor, Youyi Building Brookfields, Freetown
Ministry of Health and Sanitation

Review Comments:

- **Amendments:** Intended changes to the approved protocol such as the informed consent documents, study design, recruitment of participants and key study personnel, must be submitted for approval by the SLESRC prior to implementation.

- **Termination of the study:** When study procedures and data analyses are fully complete, please inform the SLESRC that you are terminating the study and submit a brief report covering the protocol activities. Individual identifying information should be destroyed unless there is sufficient justification to retain, approved by the SLESRC. All findings should be based on de-identified aggregate data and all published results in aggregate or group form. A copy of any publication be submitted to the SLESRC for its archive.
- **Consider Increasing your sample size to strengthen variance estimation. Title amended to reflect scope of study**

Professor Hector G. Morgan
Chair

For further enquiries please contact: efoday@health.gov.sl
