# Supplementary material for: Building confidence in crises – the roles of Sierra Leonean religious leaders’ during the 2014–2016 Ebola outbreak
Source: Glob Health Action. 2025 Sep 19;18(1):2555046. doi: 10.1080/16549716.2025.2555046 (PMC12451959; doi:10.1080/16549716.2025.2555046)
Supplement: COREQ_checklist_PL.docx [file ZGHA_A_2555046_SM1157.docx]

COREQ (Consolidated criteria for Reporting Qualitative research) Checklist

| Item No. | | Topic and Guide Questions | | Report |
| --- | --- | --- | --- | --- |
| Domain 1: Research team and reflexivity | | | |  |
| *Personal characteristics* | | | |  |
| 1 | Interviewer/ facilitator  (*Which author/s conducted the interview or focus group?*) | | PL – Lead author | |
| 2 | Credentials  (*What were the researcher’s credentials? E.g. PhD, MD*) | | MD, MSc – PL  PhD – MW, MFJ, MHA  PhD/Professor – HN | |
| 3 | Occupation  (*What was their occupation at the time of the study?*) | | All interviewers were employed as researchers at the Karolinska Institutet at the time of data collection. | |
| 4 | Gender  (*Was the researcher male or female?*) | | The research team included female and male researchers. | |
| 5 | Experience and training  (*What experience or training did the*  *researcher have?*) | | Interviewers had 2 to 15 years of previous experience in conducting qualitative research and were trained on data collection procedures and ethical considerations prior to primary data collection (*see methods chapter for further information*). | |
| *Relationship with participants* | | |  | |
| 6 | Relationship established  (*Was a relationship established prior to*  *study commencement?*) | | There was no personal relationship between interviewers and participants prior to data collection. First contact with participants was established by local NGO FOCUS1000 – page 11. | |
| 7 | Participant knowledge of the interviewer | | All interviewees were made aware that the research was a part of a masters’ dissertation with a view to academic publication. They were also aware of PL’s background as a medical doctor and researcher prior to commencement of this study. | |
| 8 | Interviewer characteristics  (*What characteristics were reported about the interviewer/facilitator? e.g. Bias, assumptions, reasons and interests in the research topic*) | | Information regarding the lead author and primary data collector, PL, is detailed on page 11 and within the strengths and limitations section on page 25 of the manuscript as well as within this checklist. This research was conducted as part of a masters thesis for PL in Karolinska Institutet. PL also has personal interest in the area of infectious disease outbreaks and health emergencies and was strongly motivated to conduct this research as a result. As part of the discussions and debriefings conducted throughout data collection and analysis, risks of biases were repeatedly discussed (*see protocol paper and supplementary file S2 for further information*). | |
| Domain 2: Study design | | |  | |
| *Theoretical framework* | | |  | |
| 9 | Methodological orientation and Theory  (*What methodological orientation was stated to underpin the study? e.g.*  *grounded theory, discourse analysis, ethnography, phenomenology, content analysis*) | | Data collection for this manuscript was guided by a thematic analysis approach with initial analysis driven by the WHO guidelines for Risk Communication in Emergencies which provided a framework for analysing risk communication in emergency settings (*see methods section for further information on data analysis processes – page 9/10*). | |
| *Participant selection* | | |  | |
| 10 | Sampling  (*How were participants selected? e.g. purposive, convenience, consecutive, snowball*) | | Religious leaders were recruited using purposive sampling, using the predefined criteria that all participants must have been involved in risk communication activities during the outbreak (*see methods section for further information on data analysis processes – page 9)*. | |
| 11 | Method of approach  (*How were participants approached?*  *e.g. face-to-face, telephone, mail, email*) | | Recruitment was conducted with the help of a local Imam who was also interviewed in the study, (*see page 9 methods for more details*). | |
| 12 | Sample size  (*How many participants were in the study?*) | | This article is based on interviews with 10 individuals (*see page 9 methods for more details*). | |
| 13 | Non-participation  (*How many people refused to participate or dropped out? Reasons?*) | | All ten of those invited to interview, attended (*see page 9 methods for more details*). | |
| *Setting* | | | | |
| 14 | Setting of data collection  (*Where was the data collected? e.g.*  *home, clinic, workplace*) | | This study was carried out in Freetown, the capital of Sierra Leone where around 1 million of the country’s 8 million people live. Eight of the ten interviews were conducted in the headquarters of FOCUS1000 (a local NGO) one took place in a local religious school and one in a pastors’ home (*see page 11 methods for more details*). | |
| 15 | Presence of non-participants  (*Was anyone else present besides the participants and researchers?*) | | Noone else was present during the interviews which took place at a time chosen by the participants and were conducted both during and after office hours (*see page 11 methods for more details*). | |
| 16 | Description of sample  (*What are the important characteristics of the sample? e.g. demographic data, date*) | | Characteristics of the sample are reported within Table 1 - Participant information on page 10 of methods section. | |
| *Data collection* | | | | |
| 17 | Interview guide  (*Were questions, prompts, guides provided by the authors? Was it pilot tested?*) | | Initial analysis was driven by the WHO guidelines for Risk Communication in Emergencies which provided a framework for analysing risk communication in emergency settings. The interview guide was largely informed by this framework and a literature search on risk communication strategies during emergencies. Questions were designed in a chronological fashion asking questions related to experiences before, during and after the outbreak. The interview started with open ended questions regarding the participant’s religious background and previous risk communication experience, continuing to questions regarding Ebola knowledge and then more targeted questions regarding experiences conducting risk communication campaigns during Ebola including barriers and facilitators to this process. Finally, open-ended questions were posed regarding reflections on the experience and thoughts going forward. Two pilot interviews were conducted leading to a few minor adjustments to question-wording and ordering before finalising the guide for the remaining interviews.  (*see page 9/10 methods section for further information*). | |
| 18 | Repeat interviews  (*Were repeat interviews carried out? If yes, how many?*) | | No repeat interviews were carried out. | |
| 19 | Audio/visual recording  (*Did the research use audio or visual recording to collect the data?*) | | All interviews were audio recorded using a sound recording device and stored anonymously on lead authors laptop. | |
| 20 | Field notes  (*Were field notes made during and/or after the interview or focus group?*) | | Brief field notes were made after each interview to summarise any thoughts the lead author relating to questions and responses. | |
| 21 | Duration  (*What was the duration of the interviews or focus group?*) | | Duration of the interviews are reported within Table 1 - Participant information on page 10 of methods section. | |
| 22 | Data saturation  (*Was data saturation discussed?*) | | Interviews were halted when recruitment was completed and 5 religious leaders from each religious background were recruited. Data saturation was achieved after 10 interviews. | |
| 23 | Transcripts returned  (*Were transcripts returned to participants for comment and/or correction?*) | | Transcripts were not returned to participants. | |
| Domain 3: analysis and findings | | | | |
| *Data analysis* | | | | |
| 24 | Number of data coders  (*How many data coders coded the data?*) | | The codebook was developed collaboratively by JW, MDCR and SAM with support of all authors. JW then coded the entire dataset while having regular debriefings with  MDCR and SAM. | |
| 25 | Description of the coding tree  (*Did authors provide a description of the coding tree?*) | | Thematic analysis was used to analyse the data, with initial open coding of three of the transcripts using both in vivo codes and codes derived by the lead author (PL). This coding framework was then applied across the remaining 7 interviews. When all transcripts had been coded, several emerging codes were retrospectively applied across the interviews and those first transcripts were revised again to check for consistency in the coding process. The codes were grouped in categories leading to the formation of sub-themes and the final themes. During the course of the analysis, authors discussed and provided feedback on the codes and the emerging themes, *see page 12 methods section.* | |
| 26 | Derivation of themes  (*Were themes identified in advance or derived from the data?*) | | In comparing and contrasting the religious leaders’ responses to one another, the analysis became more latent allowing for a deeper understanding of their opinions on their work during the outbreak allowing for the emergence of themes and sub-themes, *see page 12 methods section.* | |
| 27 | Software  (*What software, if applicable, was used to manage the data?*) | | Interviews were transcribed verbatim and analysed in Dedoose, version 8.3.4 *see page 12 methods section.* | |
| 28 | Participant checking  (*Did participants provide feedback on the findings?*) | | Findings were discussed with participants. | |
| *Reporting* | | | | |
| 29 | Quotations presented  (*Were participant quotations presented to illustrate the themes/findings? Was each quotation identified? e.g.*  *participant number*) | | We present verbatim quotes throughout the results section. We use interview number (linked to demographic information) and religious background as key identifiers. | |
| 30 | Data and findings consistent  (*Was there consistency between the data presented and the findings?*) | | We closely link our findings to our data throughout the results section. | |
| 31 | Clarity of major themes  (*Were major themes clearly presented in the findings?*) | | Major themes, as outlined in Table 2 are elaborated upon throughout the results section. | |
| 32 | Clarity of minor themes  (*Is there a description of diverse cases or discussion of minor themes?*) | | Categories and sub-themes and specific cases are discussed throughout the results section. | |

Developed based on: Tong A, Sainsbury P, Craig J. Consolidated criteria for reporting qualitative research (COREQ): a 32-item checklist for interviews and focus groups.

International Journal for Quality in Health Care. 2007. Volume 19, Number 6: pp. 349 – 357
